# Civic Education, Teaching Quality and Students’ Willingness to Participate in Political and Civic Life: Political Interest and Knowledge as Mediators

**DOI:** 10.1007/s10964-022-01639-9

**Published:** 2022-06-01

**Authors:** Pascal Alscher, Ulrich Ludewig, Nele McElvany

**Affiliations:** Center for Research on Education and School Development (IFS), TU Dortmund University, Vogelpothsweg 78, 44227 Dortmund, Germany

**Keywords:** Civic Education, Cognitive Activation, Open Classroom Climate, Political Participation, Adolescence

## Abstract

Civic education is generally assumed to play a key role in youth’s political sophistication. It aims to equip young people with the necessary competencies and skills to effectively participate in political and civic life. However, few studies have examined the relative importance of different facets of teaching quality within civic education as well as mediating factors for fostering active citizens. The present study seeks to fill this gap by investigating how different facets of teaching quality are associated with adolescents’ willingness to participate in political and civic life and how this relationship is mediated by political knowledge and interest. The study uses original data from *N* = 250 students (*n* = 152 7th graders: *M*_*age*_ = 12.54, *SD* = 0.91, range = 11–14, 45% female; *n* = 98 10th graders: *M*_*age*_ = 16.12, *SD* = 0.97, range = 15–18, 35% female). The findings show that not all teaching quality facets are equally important. While perceived cognitive activation and open classroom climate were positively associated with students’ willingness to participate, a statistically significant association with discussions of current political events in the classroom was not found. In addition, the relationship between perceived cognitive activation and willingness to participate is fully mediated by students’ political knowledge and interest. This study illustrates the relative importance of different teaching quality facets in civic education and calls for continued efforts to better understand teaching quality in civic education.

## Introduction

Democratic institutions and laws are hardly sufficient to ensure the survival of democracy (e.g., Almond & Verba 1963; Putnam, 2000). Instead, democracies and their institutions can only function well if embedded in a *culture* of democracy (see Inglehart & Welzel, 2003). Accordingly, the quality and persistence of democracy depends on the participation and underlying political sophistication of a country’s citizenry (see Milner, 2002). In general, political sophistication can refer to an individual’s political knowledge and interest. When looking at conventional forms of participation like political party engagement or voting, one can usually observe a participation gap between younger and older generations (see Maurissen, 2020). This is accompanied by worries that youth’s interests will be underrepresented in politics if younger generations participate systematically less than older generations. Such a development might threaten democracy in the long run (see Barrett, 2018). A major cornerstone within the process of political sophistication and a crucial resource for encouraging youth to participate in political and civic life is formal, school-based civic education (see Galston, 2001). Various aspects of teaching quality play an important role in civic education, offering teachers ways to engage students with political and civic content, encourage them to build political knowledge and interest, and ultimately equip them with the necessary competencies and skills to effectively participate in political and civic life. Despite the accumulating body of evidence in this field, crucial research gaps persist. Little is known about which teaching quality facets are of greatest importance in civic education. This study aims to link teaching quality through interest and knowledge with students’ willingness to participate in political and civic life. It investigates the relative importance of different civic education teaching quality facets for students’ willingness to participate and the role that political interest and political knowledge play in this relationship.

### Civic Education in Schools

Schools have a civic mission, which is to produce an informed and engaged citizenry (Campaign for the Civic Mission of Schools, 2011). School-based civic education plays an important role in fulfilling this mission (Kahne et al., 2013). It aims to equip students with the knowledge, democratic skills and attitudes necessary to effectively participate in political and civic life. It can be conceptualized as all opportunities provided by schools to engage their students in meaningful learning experiences to facilitate and enhance their development as responsible and active citizens (Homana et al., 2006). Alongside national-level and student-level aspects (see Isac et al., 2011), class-level variables like the quantity and quality of civic learning opportunities are particularly important for the success of civic education (e.g., Hess & McAvoy, 2015).

### Political and Civic Participation as a Major Outcome of Civic Education

In civic education research, it is widely accepted that political and civic participation is an integral part of a well-functioning democratic system and therefore a desired outcome of civic education. In addition to being a key defining element of democracy, political and civic participation is an expression of adolescents’ healthy and positive development (see Manganelli et al., 2015). In Germany, the standing conference of the ministers of education and cultural Affairs (*Kultusministerkonferenz*) claims that encouraging students to stand up for freedom, democracy, human rights etc. must be a vital objective of school education (Kultusministerkonferenz, 2018). Besides, attitudes towards political and civic participation usually manifest during adolescence (Baumert et al., 2016), reinforcing its position as an important outcome of civic education in schools.

The majority of political and civic participation research among adolescents is based on willingness to participate rather than actual participation. This is primarily due to adolescents’ limited opportunities and other constraints that both directly (e.g., lack of voting rights) and indirectly (e.g., lack of transportation, family responsibilities) affect their participation (McWhirter & McWhirther, 2016). Looking at intentions rather than actual behavior is based on the theory of planned behavior (Ajzen, 1991), which postulates that intentions are immediate antecedents of actual behavior. Indeed, empirical evidence from the U.S. and Italy suggests that voting intentions and voting behavior have a strong positive relationship (Ajzen et al., 1982; Roccato & Zogmaister, 2010). Willingness to participate is therefore considered the closest available proxy indicator of future political and civic participation (see Quintelier & Hooghe, 2013).

Data from the Shell Youth Studies have shown that school type matters for students’ voting intentions. More precisely, German 10th graders attending schools with an academic track have greater voting intentions than students attending schools with a vocational track (Wallrich et al., 2021). Similarly, educational attainment has a positive effect on adolescents’ voting intentions (Yang & Hoskins, 2020) and young adults at age 20 have higher self-reported actual voting levels if they completed an academic track in school compared to a vocational track (Janmaat et al., 2014).

In Germany, students are separated into different secondary tracks at the end of elementary school, which usually happens at around age 10. The school assignment procedure is primarily based on students’ achievements and abilities. Although there is considerable variation across German states, the most common system is the “tripartite” system of Hauptschule, Realschule and Gymnasium. Among the three, Hauptschule is the most vocationally oriented track. Realschule is the intermediate track and Gymnasium is the academically most challenging track. For clarity, the study differentiates between schools that prepare for vocational education and training (i.e. Hauptschule and Realschule) and Gymnasium, which prepares students for university entrance. Usually, the proportion of students from immigrant backgrounds is higher and average socio-economic status lower among students in schools preparing for the vocational track (Wallrich et al., 2021). In addition, research has shown that students in the vocational track have less access to civic education classes (Achour & Wagner, 2020).

Furthermore, research suggests that adolescents’ willingness to participate in political and civic life depends on their socio-economic background. For instance, in the Italian subsample of the ICCS 2009 data, the national index of socio-economic background, comprising parents’ highest occupational status, parents’ highest educational level, and the number of books at home, is significantly positively associated with students’ expected electoral expectation (Manganelli et al., 2012). This result was replicated when considering political knowledge and efficacy beliefs as mediating variables (Manganelli et al., 2014). However, empirical evidence suggests that effective civic education through high teaching quality can weaken the link between socio-economic background and willingness to participate (e.g., Castillo et al., 2015).

Despite some national differences, most countries’ education systems share a rather broad concept of citizenship (Claes & Hooghe, 2009). In Western European countries, teacher beliefs regarding the aims of civic education are very similar (Reichert & Torney-Purta, 2019). In addition, ICCS 2016 data show that the relationship between civic education and civic outcomes is rather stable across countries. In all 24 participating countries, political interest and knowledge were significantly positively associated with students’ willingness to participate in.

### Teaching Quality in Civic Education

Teaching quality has a long tradition in educational research and has proven to be a relevant feature of schools’ effectiveness (see Hattie, 2012). In general, teachers play a crucial role in determining how civic education is taught. Not only are teachers able to adjust the content of civic education classes within the outlines of the official curriculum to students’ needs, they can also adjust their classroom behavior.

From the perspective of deliberative democracy theory, which stresses the importance of discussion and deliberation in democracy (see Goodin & Spiekermann, 2018), an important facet of teaching quality in civic education is an open classroom climate—that is, a classroom climate in which controversial issues are openly discussed with respect for everyone’s opinion (Godfrey & Grayman, 2014). A multitude of studies have shown that adolescents in classes with frequent and open deliberation perform better on various civic outcomes (e.g., Campbell, 2008; Eckstein et al., 2021; Manganelli et al., 2015). Data from the International Citizenship and Education Study (ICCS) 2016 revealed that in all participating countries, students’ perceptions of an open classroom climate were on average positively associated with students’ political knowledge and political interest (Schulz et al., 2018). Similar findings were reported for predecessor studies (Schulz et al., 2009; Torney-Purta et al., 2001).

Another relevant facet of teaching quality in civic education is the discussion of current political events in the classroom. Deliberating about current political controversies and events provides opportunities for students to consider diverse perspectives and develop political opinions (McCafferty-Wright & Knowles, 2016). Furthermore, it equips students with skills to engage with the political discourse and democratic life beyond school (Hess & McAvoy, 2015) and enables them to gain political knowledge apart from their textbooks. In particular, exposing students to current political controversies might spark their political interest. Based on data from the 2010 NAEP 12^th^ grade Civics Assessment, it could be shown that while controlling for students’ demographic characteristics and aspects of their home environment, talking about current political events at any time in class was positively associated with scores on the NAEP Civics Assessment (Bittman & Russel, 2016). Moreover, data from the California Survey of Civic Education, comprising 2,366 California high school seniors shows that 61% of students who reported frequent talk about current political events in class said that they were interested in politics, compared to only 32% of students who reported no talk about current political events in class (Kahne, 2005).

Another facet of teaching quality in civic education that has been rather overlooked so far is cognitive activation. Cognitive activation describes teachers’ abilities to create learning activities with a greater potential for higher-level thinking, such as the provision of appropriately challenging teaching material, capitalization on students’ prior knowledge and grappling with the learning content in an in-depth way (e.g., Stang & McElvany, 2020). In civic education particularly, cognitive activation could refer to encouraging students to shift their perspective and review political decisions regarding their argumentative robustness. Furthermore, cognitively activating tasks equip students with collective problem solving skills. Cognitive activation has proven to be an effective tool for stimulating students’ learning and interest in other subjects (e.g., mathematics: Baumert et al., 2010; science learning: Fauth et al., 2014; Biology: Förtsch et al., 2016), while empirical evidence about its potential role in civic education is limited. But some initial results from Germany suggest that perceived cognitive activation has a positive effect on 9th and 10th graders’ civic education-specific content knowledge (Weißeno & Landwehr, 2015).

The exact relationship between civic education teaching quality and willingness to participate has not yet been well researched. Theoretically, two processes are conceivable as to how teaching quality is related to willingness to participate. The first possibility is that teaching quality is directly related to willingness to participate. Theoretical considerations regarding a direct relationship are scarce. One possible explanation could be that increased teaching quality leads to increased expectations regarding one’s own behaviors. However, there seems to be no empirical evidence to support such a direct relationship. The second possibility is that civic education teaching quality is only indirectly related to willingness to participate. The relationship between civic education and willingness to participate might be mediated by more immediate goals of civic education, such as political knowledge and political interest (see Bayram-Özdemir et al., 2016; Maurissen, 2020).

### Political Knowledge and Political Interest as Mediators

Raising students’ political knowledge and stimulating their political interest are essential immediate goals of civic education instruction (Claes & Hooghe, 2008). At the same time, students’ political knowledge and political interest are of crucial importance in determining voting habits (e.g., Gil de Zúñiga & Diehl, 2019; Grobshäuser & Weißeno, 2020) and predicting other political and civic activities (e.g., Quintelier & Hooghe, 2012; Reichert, 2016). Therefore, civic education teaching quality may be indirectly associated with students’ willingness to participate via political knowledge and political interest (Maurissen, 2020). A distinction between different immediacies of civic education goals is similarly reflected in moral development and character education theory. On the one hand, traditional or direct citizenship education explicitly focuses on knowledge transfer, which can facilitate the subsequent acquisition of other political and participation skills. On the other hand, indirect civic education places less emphasis on knowledge acquisition and focuses on moral reasoning and democratic values, thus attitudes and intentions (Clase & Hooghe, 2009). So far, these considerations have remained limited to the question of what citizenship norms civic education should pursue. This study expands their application by integrating the two approaches and defining more immediate (i.e. political interest and knowledge) and less immediate (i.e. willingness to participate) civic education outcomes.

Political knowledge involves “the range of factual information about politics that is stored in long-term memory” (Carpini & Keeter, 1996, p. 10). It is a crucial immediate goal of civic education and it relates to political and civic participation. Usually, students’ knowledge gains are evaluated with standardized tests, which enable teachers to directly evaluate students’ progress. Furthermore, political knowledge has repeatedly proved to be an important prerequisite for political and civic participation (e.g., Grobshäuser & Weißeno, 2020). Even a little knowledge about politics can empower citizens to make meaningful and informed decisions by virtue of judgment heuristics (Niemi & Junn, 1998).

Political interest is another important and immediate goal of civic education and a prerequisite for political and civic participation. Empirical evidence suggests that motivation for learning based on interest stimulates the learning process and positively affects learning outcomes (Krapp 2002). Accordingly, stimulating students’ (domain-specific) interest is of crucial importance for scholastic achievement. Many studies suggest that political interest usually manifests during youth and changes little afterwards (Baumert et al., 2016). This further stresses the role of schools and civic education in particular in the process of stimulating youth’s political interest. Moreover, it is a well-established notion that political interest is an effective prerequisite for meaningful political and civic participation as well as for gathering political information (e.g., Claes & Hooghe, 2017; Prior & Bougher, 2018).

## Current Study

Enabling young people’s effective participation in political and civic life in modern-day democracies must be an essential objective of education, and of civic education in particular. Despite the growing amount of research in the field, gaps still exist concerning the relative importance of different facets of teaching quality and the relationship between civic education and students’ participation in political and civic life. First, this study examines how perceptions of (i) an open classroom climate, (ii) discussions of current political events, and (iii) cognitive activation within civic education are associated with students’ willingness to participate in political and civic life. It is hypothesized that all three facets are positively related to willingness to participate even when considered simultaneously. In addition, the relative importance of each facet will be explored. Second, this study investigates the question of how the relationship between these perceived teaching quality facets and willingness to participate is mediated by (i) political knowledge and (ii) political interest. It is hypothesized that the relationships between the perceived teaching quality facets and students’ willingness to participate are mediated by political knowledge and political interest.

## Methods

### Participants

The data for this research project was collected in fall 2020 in the Study on the Development of Political and Civic Competence in Adolescence (German: *Studie zur Entwicklung politischer und gesellschaftlicher Kompetenz im Jugendalter, EPKO*). The participating students were attending 7th or 10th grade. In some German federal states, students enter secondary education in 7th grade, whereas in most others states, secondary education begins in 5th grade. In addition, students in the vocational track leave school after the 10th grade and typically enter vocational training. The period from 7th to 10th grade thus marks the core of secondary education in Germany. The schools were located in the two federal states of North Rhine-Westphalia and Thuringia. A total of 17 classes with *N* = 250 students (*n* = 152 7th graders: *M*_*age*_ = 12.54, *SD* = 0.91, range = 11–14, 45% female; *n* = 98 10th graders: *M*_*age*_ = 16.12, *SD* = 0.97, range = 15–18, 35% female) participated in the study. The response rate was 73%. Students not reached either did not have informed consent or were absent on the test day due to illness or quarantine measures. According to the curricula, almost all participating students had already received civic education classes as part of so-called contingent lessons (*Kontingentstunden*), which allow schools to determine the exact scope and timing of the classes themselves. Furthermore, the exact amount of instructional time varies across federal states and school types. Therefore, and acknowledging the interdisciplinary character of civic education, students were asked to answer the teaching quality instruments with regard to all classes in which they talk about political and civic affairs. A majority of participants (*n* = 164) were enrolled in an academic-track school (i.e. *Gymnasium*). The proportion of participants with an immigrant background was with 31.6% a little lower than in a comparable German study with 4^th^ graders (Ohle-Peters et al., 2021, Mig. = 38.0%). In Germany, students have different teachers for different subjects. In this particular sample, students were part of a stable group of peers that remained intact across subjects.

This study was conducted in compliance with the German Research Foundation’s guidelines for good scientific practice (*Deutsche Forschungsgemeinschaft, DFG*). In addition, the University Joint Ethics Committee examined and approved the research project with regard to protection of human dignity and the autonomy and self-determination of the people involved in the research. Prior to data collection, all participants and their parents were asked to give their informed consent. Each class was awarded 50 EUR for their participation.

### Measures

#### Outcome: Willingness to Participate in Political and Civic Life

In order to assess their willingness to participate in political and civic life, students were given eleven items describing political and civic activities. Item selection was mainly driven by Ekman and Amnå’s (2012) typology of political participation and prior empirical evidence (e.g., Albert et al., 2019; Gaiser et al., 2016). The introductory statement read as follows: “If you want to voice your opinion, how likely are you to take the following actions?” For each item, students chose a response on a four-point scale comprising 0 = “not at all likely”, 1 = “rather not likely”, 2 = “rather likely”, and 3 = “very likely”. Each item referred to a different political or civic activity. Thus, responses on each item were summed up to form an index ranging from 0 to 33, with higher scores indicating higher willingness to participate in more political and civic activities.

#### Teaching Quality Facets

In Germany, depending on grade level, federal state and school type, civic education classes differ with regard to their name, focus, degree of integration and position in the curriculum. In order to avoid misunderstandings, students were asked to answer the questions regarding teaching quality facets with respect to all situations in which they talk about civic or political affairs in class. Thus, students’ perceptions of teaching quality cover but are not limited to civic education classes.

#### Open Classroom Climate

To capture students’ perceptions of the frequency with which teachers created an open classroom climate, the German translation of the well-established scale from ICCS 2016 was used, e.g., “Teachers encourage students to make up their own minds” (Schulz et al., 2018). Specifically, students were provided with an introductory question asking “How often do you experience the following situations in class?” Students were then asked to rate each item on a four-point scale comprising 1 = “never”, 2 = “rarely”, 3 = “sometimes”, and 4 = “often”. In line with prior theoretical considerations and empirical evidence, the four items related to teachers’ behaviors were used and the two items focusing on students’ behaviors were disregarded. The scale’s overall Cronbach’s alpha reliability was 0.79 (0.80 and 0.76 for 7th and 10th graders, respectively). For mean, standard deviation and correlations see Table 1.

**Table 1:** Means, standard deviations, and correlations

Variable	1	2	3	4	5	6	7	8	9	10	11
1. Willingness to Participate											
2. Political Interest^a^	0.41**										
3. Political Knowledge	0.33**	0.37**									
4. Open Clasroom Climate^a^	0.33**	0.08	−0.01								
5. Cognitive Activation^a^	0.39**	0.31**	0.20**	0.44**							
6. Discussion of Current Political Events	0.08	0.28**	0.10	0.30**	0.26**						
7. School Type^b^	0.32**	0.02	0.27**	−0.02	0.07	−0.36					
8. Grade^c^	−0.02	0.15*	0.42**	0.09	0.01	0.38**	−0.38**				
9. Cultural Capital	0.28**	0.16*	0.40**	0.02	0.02	−0.09	0.61**	−0.09			
10. Immigrant Background^d^	−0.09	−0.14*	−0.29**	−0.03	−0.09	0.08	−0.42**	0.09	−28**		
11. Math Grade	0.07	0.08	0.27**	0.08	0.06	−0.12	0.27**	−0.04	−0.24**	−0.13	
*M*	16.65	2.12	0.30	2.85	2.58	2.62	0.66	0.39	3.55	0.32	4.14
*SD*	6.52	0.73	0.90	0.70	0.76	0.86	0.48	0.49	1.65	0.47	1.06
*N*	204	238	250	234	225	247	250	250	247	247	204
Range	0–33	1–4	−2.4–4.6^e^	1–4	1–4	1–4	0–1	0–1	1–6	0–1	1–6

*Note. M* and *SD* represent mean and standard deviation, respectively.

^a^*M* and *SD* are calculated using the row means of the variable indicators.

^b^0 = school without academic track and 1 = school with academic track

^c^0 = 7th grade and 1 = 10th grade

^d^0 = no immigrant background and 1 = immigrant background

^e^rounded to one decimal place

*p < 0.05; **p < 0.01

#### Discussion of Current Political Events

Perceived frequency of discussions of current political events in class was measured with a single item asking “How often do you talk about current political events in class?” (see e.g., Bittman & Russell, 2016). Again, responses were given on a 4-point scale from 1 = “never” to 4 = “often”.

#### Cognitive Activation

Perceived frequency of cognitive activation in class was measured with three items which were adopted from the COACTIV Study (Baumert et al., 2008, p. 129) and adjusted to the needs of this study. The items were preceded by the question “How often do you experience the following situations?” The three items were (1) “The teacher asks questions that cannot be easily answered, but which stimulate our thinking”, (2) “The teacher gives us tasks for which the primary goal is not to find the right solution but to find good arguments for our answer.”, and (3) “The teacher gives tasks for which we have to discuss with each other.” Each of the three items was rated on a four-point scale ranging from 1 = “never” to 4 = “often”. The scale’s overall alpha reliability was 0.79 (0.81 and 0.76 for 7th and 10th graders, respectively).

#### Mediator A: Political Knowledge

To measure students’ political knowledge, an elaborate instrument was developed to match the skills of students in the 7th and 10th grade and the idiosyncrasies of the German political system (Alscher et al., 2022). Questions with different item difficulties were selected to represent the broad spectrum of political knowledge. A total of 99 items were used. The items were organized around the three content domains (1) political structures, (2) political processes, and (3) political principles dealing with different aspects of political and civic life, such as parliament, voting and human rights. Each item was a forced-choice question with three wrong and one correct answer option. Correct answers were coded as 1 and incorrect answers were coded as 0.

The 99 items included 36 grade-specific items for 7th and 10th grade as well as 27 anchor items answered by both 7th and 10th graders, resulting in a data structure known as a non-equivalent groups anchor test design (Paek & Cole, 2020). The difficulty of the items was approximated in advance of the surveys by a panel of experts consisting of professors of civic education instruction and study coordinators of large-scale assessment studies. Grade-specific items were assigned based on the difficulty levels determined by the experts. As students completed 63 items each, a maximum score of 63 points could be reached.^1^ To link the test scores from 7th and 10th graders, a unidimensional multigroup 1-parameter model was calculated using the “TAM” package (Robitzsch et al., 2021) in R. Before calibration, rapid-guessing behavior was detected based on students’ response times. In accordance with a widely accepted approach, the bimodality of the response time distribution was used to visually identify the threshold at the local minimum between the two modes (Wise, 2017). For this test, this threshold was set slightly above the visual low point, at 6.5 seconds, leading to the deletion of 4.48% of answers. Including not-reached items, a total of 5.71% of responses were missing. During calibration, the estimated individual ability (θ) was calculated, ranging from −2.39 to 4.56, with higher scores representing more political knowledge. The WLE reliability was 0.88. For test security reasons, nine versions of each test, differing only in the order of the items, were administered.

#### Mediator B: Political Interest

Political interest was measured with the Short Scale Measuring Political Interest (SSPI: Otto & Bacherle, 2011). The scale includes five items, e.g., “I observe political events with great interest.” The introductory question read as follows: “To what extent do the following statements apply to you?” The students were asked to rate each item on a four-point scale ranging from 1 = “doesn’t apply at all” to 4 = “applies very well”. The scale’s overall alpha reliability was 0.89 (0.87 and 0.91 for 7th and 10th graders, respectively).

#### Control Variables

Cultural capital was operationalized by the reported number of books in the student’s home (adapted from PISA 2015, see Mang et al., 2019). Responses ranged from 1 = “0 to 10 books” to 6 = “more than 500 books”. In addition to the numbers, each answer option was accompanied by a picture of a shelf displaying the relevant number of books. For immigrant background, students were asked which language they speak at home (adapted from IGLU 2016, see Hußmann et al., 2020). For those students who reported to speak at least sometimes a language other than German at home, an immigrant background was assumed.

Furthermore, 7th grade was coded as 0 and 10th grade as 1. In terms of school type, schools preparing for a vocational track were coded as 0 and schools preparing for an academic track were coded as 1. For school achievement, students’ self-reported math grade ranging from 1 = “inadequate” to 6 = “very good” was used.

### Procedure

The study was administered in the first three class periods of a regular school day in the students’ classrooms via computer-based assessment. After distributing the laptop computers to the students, trained research assistants explained the study procedure and the goals of the research project to the students. The study consisted of four parts: (1) a 60-minute political knowledge test, (2) a 20-minute cognitive ability test, (3) a 10-minute reading comprehension test, and (4) a 35-minute questionnaire. After each section, students were given short breaks. They could only resume their participation when the test coordinator provided them with the necessary keyboard shortcut to move on to the next screen on their laptop computers. After the data collection, students were given the opportunity to voice immediate feedback that will be used in future studies.

### Data Analysis

All analyses were conducted using structural equation modeling and the “lavaan” package in R. The first structural equation model included the predictor variables (i.e., the civic education teaching quality facets) and the main outcome variable (i.e., students’ willingness to participate in political and civic life). In the second model, the aforementioned control variables (i.e., cultural capital, immigrant background and school variables) were included as predictor variables to check the robustness of the results. In the third model, the mediating variables were added (i.e., political interest and political knowledge), while still enabling direct associations between the perceived civic education teaching quality facets and willingness to participate. In all models, open classroom climate, cognitive activation and political interest were operationalized as latent variables. All paths between the predictors, mediators and outcomes were specified. However, for the second model, only significant paths are displayed to declutter the figure. Intercorrelations were enabled between all exogenous variables and between the two mediating variables. For clarity, the intercorrelations of the control variables are not reported in the figures. A one-way random-effect analysis of the intra-class correlation revealed that only 4, 6 and 5% of the variance in open classroom climate, cognitive activation and discussion of current political events, respectively, are explained by the class structure in the sample. Together with the small sample size, this led to the refusal of multilevel analysis.

We used the full information maximum likelihood (FIML) method to estimate the models with missing data. Moreover, the factor loadings of all latent variables’ indicators were fixed to 1. Evaluation of the models’ goodness of fit was based on the comparative fit index and Tucker-Lewis index (CFI ≥ 0.95 and TLI ≥ 0.90) as well as the root mean square error of approximation (RMSEA < 0.08) and the standardized root mean square residual (SRMR < 0.08) (Hu & Bentler, 1999). Furthermore, the relative importance of the three civic education teaching quality facets was tested in both models by imposing equality constraints for the otherwise free parameters of the teaching quality facets. Afterwards, the model fits of the models with and without equality constraints were compared.

## Results

### Descriptive Statistics

The mean score for cultural capital (i.e., the number of books in the students’ home) was a little higher than in comparable German studies (e.g., Heyder et al., 2021; *M* = 2.88) at *M* = 3.55. This could be explained by the marginal overrepresentation of students from higher-track schools in the sample. Average scores for the perceived civic education teaching quality facets were slightly above the midpoint of the response scale. This is in line with the German ICCS 2016 data (Abs & Hahn-Laudenberg, 2017) for perceived open classroom climate and with the 2010 NAEP data (Bittman & Russell, 2016) for perceived frequency of discussions of political events. The mean score for political interest, *M* = 2.17, is lower than the item-specific mean scores reported in the original pilot studies (*M* = 2.50 to 3.51, Otto & Bacherle, 2011). However, only adults (*M*_age_ = 38.6 and 36.5) participated in these pilot studies, which presumably explains the higher average level of political interest. Comparable studies investigating German secondary education students’ willingness to participate in political and civic life (Weißeno & Landwehr, 2018; Weißeno & Schmidt, 2019) have found that students demonstrate a moderate level of willingness to participate in political and civic life, which was in line with this study’s results. The bivariate correlations in Table 1 show that the three facets of civic education teaching quality are statistically significantly, yet only moderately correlated with each other (0.25 to 0.36).

### Associations Between Civic Education Teaching Quality and Students’ Willingness to Participate in Political and Civic Life

**Fig. 1:**
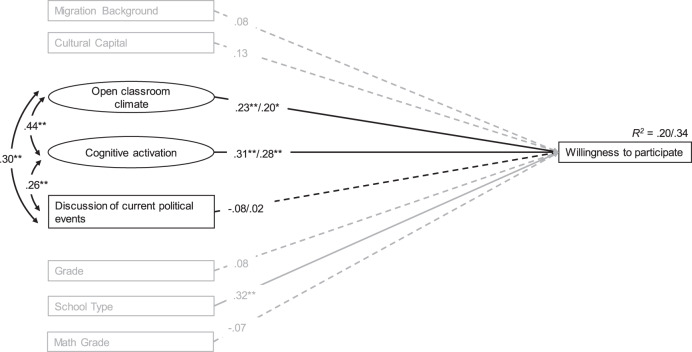
Direct associations between civic education teaching quality facets and willingness to participate. Note: Values on the left are estimates without control variables, values on the right when control variables are included. Significant paths are solid; nonsignificant paths are dashed. CFI: 0.953/0.955; TLI: 0.939/0.923; RMSEA: 0.062/0.054; SRMR: 0.052/0.043. *p < 0.05; **p < *0.01*

The bivariate correlations show that perception of an open classroom climate and perceived cognitive activation are statistically significantly associated with students’ willingness to participate in political and civic life (0.31 and 0.35 respectively). Only the perceived frequency of discussions of current political events is not statistically significantly associated with students’ willingness to participate. To examine how these associations might shift when considering all three facets simultaneously, a partially latent structural equation model was fitted examining the direct associations between the perceived civic education teaching quality facets and students’ willingness to participate. Looking at the standardized path coefficients (see Fig. 1), one sees that the perceived frequency of an open classroom climate and cognitive activation are indeed statistically significantly positively associated with students’ willingness to participate in political and civic life (*ß* = 0.23 and 0.31, *p* = 0.007 and 0.000 respectively). However, the model did not reveal a direct association between perceived frequency of discussions of current political events and students’ willingness to participate (*ß* = −0.08, *p* = 0.261). For the control variables, all associations with the outcome variable were in the expected direction, although only the association between school type and students’ willingness to participate was statistically significant (ß = 0.32, p = 0.001). The inclusion of the control variables led to only minor changes in the magnitude of the coefficients and little altered the significance levels. Overall, the model explained 20% of the variance in the outcome variable, and 34% when including the control variables. For testing the relative importance of the teaching quality facets, the model that includes the control variables was used. The test showed that cognitive activation is a significantly stronger predictor for students’ willingness to participate than the discussions of current political events. The chi-square test revealed that the model with equality constraints fits the data significantly worse than the model without equality constraints (*p* = 0.020). When comparing the importance of open classroom climate and the discussion of current political events, the chi-square test failed to meet the 5% significance threshold (*p* = 0.080). Moreover, cognitive activation was not a significantly stronger predictor than open classroom climate (*p* = 0.686).

### Political Knowledge and Political Interest as Mediators between Teaching Quality and Students’ Willingness to Participate in Political and Civic Life

**Fig. 2:**
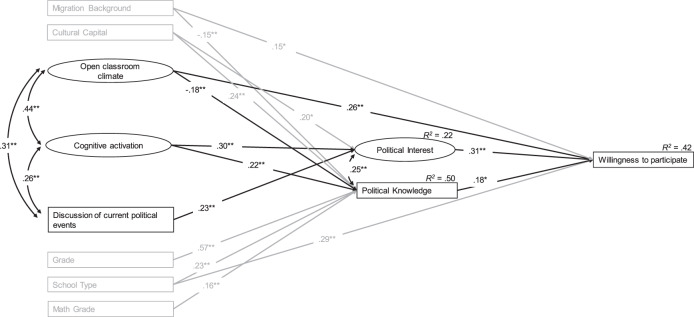
Associations between civic education teaching quality facets and willingness to participate mediated by political interest and political knowledge. Note: Only significant paths are displayed. CFI: 0.934; TLI: 0.910; RMSEA: 0.059; SRMR: 0.064. *p < 0.05; **p < *0.01*

To answer the second research question, a model was fitted examining the underlying mechanism of the associations between the civic education teaching quality facets and students’ willingness to participate. For this purpose, political interest and political knowledge are included as mediating variables in the model (see Fig. 2). As expected, both political interest and political knowledge were statistically significantly positively associated with students’ willingness to participate (*ß* = 0.31 and 0.18, *p* = 0.000 and 0.029 respectively). Perceived frequency of an open classroom climate is still directly positively associated with willingness to participate (*ß* = 0.26, *p* = 0.001). Rather counterintuitively, the perceived frequency of an open classroom climate is statistically significantly negatively associated with students’ political knowledge (*ß* = −0.18, *p* = 0.006). Perceived frequency of cognitive activation is statistically significantly positively associated with both political interest (*ß* = 0.30, *p* = 0.001) and political knowledge (*ß* = 0.22, *p* = 0.001). The direct association between perceived frequency of cognitive activation and willingness to participate observed in the first model is now statistically significantly mediated by students’ political interest (*ß* = 0.09, *p* = 0.006). However, the mediation via political knowledge fails to meet the 5% significance threshold (*ß* = 0.04, *p* = 0.067). The total indirect association is statistically significantly positive (*ß* = 0.13, *p* = 0.001). Furthermore, perceived frequency of discussion of current political events is positively associated with political interest. The three education-related control variables grade, school type and math grade are associated with students’ political knowledge but not with their political interest. Overall, this model explains 42% of the variance in willingness to participate, 22% of the variance in political interest and 50% of the variance in political knowledge. The findings suggest that perceived frequency of an open classroom climate is directly associated with students’ willingness to participate and not mediated by political knowledge or interest, while the association between perceived frequency of cognitive activation and students’ willingness to participate is fully mediated by political interest and partially by political knowledge. Discussion of current political events is positively associated with political interest but neither directly nor indirectly associated with students’ willingness to participate. To test for the relative importance, the total effects of teaching quality facets, thus both indirect effects through political interest and political knowledge as well as the direct effect, on students’ willingness were compared. The chi-square test showed that cognitive activation is an overall stronger predictor than the discussion of current political events (*p* = 0.018). For the comparison of the open classroom climate and the discussion of current political events, the chi square test barely missed the 5% significance threshold (*p* = 0.057). Regarding the importance of cognitive activation and open classroom climate no significant differences were found (*p* = 0.758).

### Sensitivity analyses and alternate model analyses

**Fig. 3:**
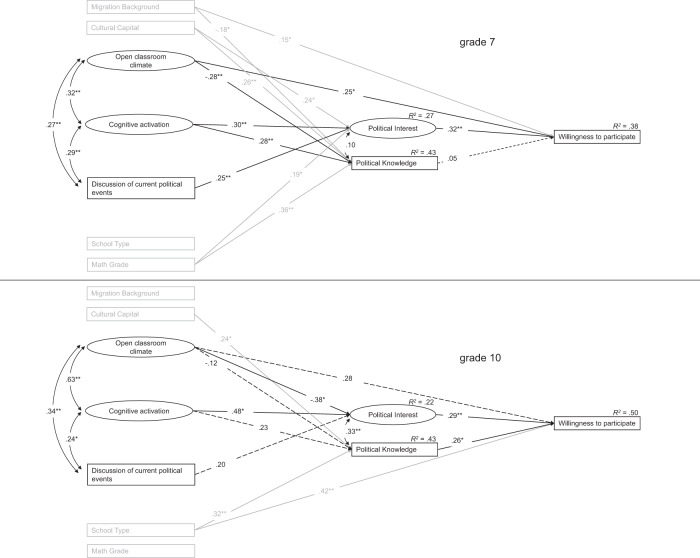
Associations in 7^th^ and 10^th^ grade between civic education teaching quality facets and willingness to participate mediated by political interest and political knowledge. Note: Only significant paths are displayed. K7: CFI: 0.925; TLI: 0.901; RMSEA: 0.060; SRMR: 0.076.; K10: CFI: 0.959; TLI: 0.943; RMSEA: 0.050; SRMR: 0.074. *p < 0.05; **p < *0.01*

Additional analyses were carried out to confirm the robustness of the findings. Two separate analyses were run for participants from the 7th and the 10th grade. The overall pattern of findings remained intact. Numerically, school type plays a bigger role in the 10th grade while cultural capital and immigrant background are less important. The explained variance in students’ willingness to participate was a little higher in the 10th than in the 7th grade. Regarding the civic education teaching quality facets, no systematic changes were observed. However, statistical power is limited due to the reduced sample sizes (Fig. 3).

## Discussion

Civic education is generally thought to be an important tool in educating youth to become responsible, informed and active citizens (e.g., Neundorf et al., 2016). In particular, civic education teaching quality facets and students’ perceptions thereof play a crucial role in the achievement of desired civic outcomes among youth (Bayram-Özdemir et al., 2016; Manganelli et al., 2015). This is even more important in light of the limited time available for civic education in German schools’ curriculum (Hedtke et al., 2021). However, previous research has usually focused on a single teaching quality facet, thus failing to test the relative importance of different teaching quality facets in civic education. Testing the relative importance matters because it enables us to better understand the contribution of different predictors to the variance in an outcome, unraveling the relative strength of these predictors. Moreover, whereas research on an open classroom climate and discussion of current political events have a long tradition in civic education research, cognitive activation has been largely overlooked. In addition, only little previous research has explored the mechanism underlying the relationship between civic education and willingness to participate by including mediating variables that are more immediate goals of civic education and function as prerequisites for political and civic participation.

The results show that a perceived open classroom climate and perceived cognitive activation are positively associated with students’ willingness to participate. Students’ perceptions regarding the frequency of discussions of current political events, however, are not associated with willingness to participate. The findings therefore only partially confirm the first hypothesis which assumes that all three facets have a positive relationship with willingness to participate. There is reason to believe in the robustness of these results, since the described associations remained after including control variables. The results regarding the relative importance of the facets further stress the importance of cognitive activation and open classroom climate.

Political knowledge and political interest mediated the association between perceived cognitive activation and willingness to participate, suggesting that perceived cognitive activation plays an important role for the achievement of these more immediate goals of civic education. However, perceived open classroom climate still has a direct relationship with willingness to participate. Therefore, the findings again only partially confirm the second hypothesis. One reason for the continued direct relationship might be that the relationship is mediated through another more immediate outcome of civic education, such as political efficacy (see Bayram-Özdemir et al., 2016; Maurissen, 2020). Another explanation could of course be that an open classroom climate is indeed directly associated with willingness to participate. Future research should further investigate the mechanism underlying this relationship.

Furthermore, discussing current political events seems to be an important contributor to students’ political interest, but not to their political knowledge. This finding is counterintuitive, at least given that previous findings suggest that the discussion of current political events is associated with increased political knowledge (Bittman’s & Russel’s, 2016). However, unlike this study, Bittman and Russel (2016) did not control for an open classroom climate and cognitively activating teacher behavior. Data from the Civic Education Study in the United States in 1999 revealed that Traditional Teaching methods, including heavy usage of textbooks, memorization, worksheets, reports, extra materials and discussion of current political events has a statistically insignificant negative relationship with political knowledge (Martens & Gainous, 2013). However, the study also revealed that in combination with an open classroom climate, traditional teaching is the most effective overall pedagogical approach to civic education. One possible explanation for these inconsistent and sobering findings regarding the discussion of current political events in the classroom could be that teachers have varying time resources to prepare and lead discussions on current political events in a way that provides for meaningful learning opportunities (Kawashima‐Ginsberg & Levine, 2014).

Surprisingly, the results show that when multiple teaching quality facets are considered a perceived open classroom climate is negatively associated with students’ political knowledge. One possible explanation for this finding could be that more knowledgeable students have increased expectations regarding an open classroom climate and are therefore more often disappointed with actual classroom discussion conditions. A very open classroom might also come at the expense of structure and therefore hinder knowledge transfer. Another explanation might be that students who are very committed to academic achievement might feel a strong imperative to endorse their teacher’s opinions and class material and hence, perceive the classroom to be less open. The bivariate correlation suggests no relationship between open classroom climate and political knowledge.

Other recent research also applies a more critical approach towards the assessment of open classroom climate. For instance, an open classroom climate alone does not ensure a nurturing social context in diverse classrooms (Munniksma et al., 2021). In contradiction of previous findings (e.g., Campbell 2008; Eckstein & Noack, 2015), an open classroom climate might not be effective in reducing social inequalities in political engagement (Deimel et al., 2020; Hoskins et al., 2017). Moreover, a video study of nine 8th and 9th grade civic education classes in Germany, showed that open classroom discussions work differently depending on students’ personality traits (Gronostay, 2019). For instance, agreeableness was negatively and extraversion positively associated with students’ participation in class discussions. However, it should be mentioned that other recent studies did find positive, statistically significant associations between students’ perceptions of an open classroom climate and political knowledge (e.g., Carrasco et al., 2020; Sampermans et al., 2020).

Moreover, the explained variance in students’ willingness to participate in political and civic life nearly doubles in the second model compared to the first. This finding stresses the added value generated by introducing the more immediate goals of civic education as mediators. It should further be noted that the relationship between civic education teaching quality facets and political knowledge might be underestimated in this study. The knowledge test developed for this study was not tied to the curriculum and contains content that is learned outside of civic education classes. Hence, political learning outside of civic education classes and school might be more influential than it would be in a test that is tied to the curriculum. The results nonetheless suggest that political knowledge and political interest might be crucial intermediates in the relationship between civic education teaching quality and students’ willingness to participate.

Despite its strengths, this study also comes with a few limitations. The data was collected in fall 2020, shortly before the second pandemic-driven lockdown in Germany, in which schools were closed entirely. The small sample size makes it impossible to account for the hierarchical structure of the data, since multilevel models did not converge. The low intra-class correlations indicate that students within a particular class evaluate teaching quality in their class differently. However, these findings are line with previous research (e.g., Praetorius et al., 2017) and might be explained by differences in perceptions or differences in actual treatment by the teacher. Future research should address the extent to which student perceptions, teacher statements, and objective measures (e.g., ratings by external observers) of instructional quality are interrelated and influence civic education outcomes. Furthermore, students were asked to answer the relevant questions with regard to all classes in which they talk about political and civic affairs, as not all students had dedicated civic education classes at the time data collection. This might underestimate the role of civic education instruction. It must be acknowledged that there may be discrepancies between students’ perceptions of civic education teaching quality facets and the actual levels of these teaching quality facets. Ideally, future studies add to the literature by using independent measures of civic education teaching quality facets, such as systematic class observations. Although previous studies have used a single-item solution to measure discussion of current political events (see Bittmann & Russel, 2016) and the corresponding findings yield plausible relationships, future studies should use a scale instead. Eventually, future research should aim for a better understanding of the relationship between an open classroom climate and students’ political knowledge. There may also be additional facets of teaching quality in civic education that are not considered in this study, as well as other contextual influences, like participation in extracurricular civic activities, students’ home environment, and students’ peers. This study’s strengths include its incorporation of multiple civic education teaching quality facets, making it possible to assess the relative importance of these facets. In addition, some of the instruments were newly developed in an elaborate, rigorous process and offer novel opportunities to measure perceived cognitive activation in civic education and political knowledge.

The results bear important implications for educational practice and policy. Due to their distinct mode of action, specific attention should be paid to different facets of teaching quality and their role in schools’ fulfillment of their civic mission. Focusing on the creation of an open classroom climate alone might be insufficient or even detrimental for students’ knowledge acquisition. Furthermore, vocational track students’ disadvantageous position with regard to important civic outcomes warrants attention. There is thus a need for German educational policy to address the problem of systematic disparities in the formal qualification of civic education teachers across school tracks which might be a source of this difference.

## Conclusion

While in recent years, teaching quality in civic education has ranked highly on the scholarly agenda, the relative importance of different teaching quality facets remains understudied. To better understand teaching quality in civic education, the current study investigated three different teaching quality facets and their relationship with students’ willingness to participate in political and civic life, their political knowledge and their interest. The results suggest that not all perceived teaching quality facets are equally important for students’ willingness to participate. In particular, fostering and stimulating student engagement through cognitive activation seems to play a bigger role than previously assumed. In addition, the relationship between perceived cognitive activation and willingness to participate is mediated by political interest and political knowledge. Future research should continue to strive to better understand how teaching can improve civic education and contribute to schools’ mission to produce responsible, informed and active citizens.

## Footnotes

1 For test development, we relied upon an advisory committee of experts, consisting of renowned German professors of civic education and the director of an international large-scale assessment of civic education and civic competence. The experts had prior experience with the development of knowledge tests. In a first phase, the experts were invited for a one-day workshop to talk about how the knowledge test framework could be conceptualized. In a second phase, the experts rated all preliminary items for plausibility, relevance, difficulty, and relationship to the knowledge test framework. Furthermore, they were given the opportunity to comment on the items in an open-ended way.
